# Bevacizumab promotes venous thromboembolism through the induction of PAI-1 in a mouse xenograft model of human lung carcinoma

**DOI:** 10.1186/s12943-015-0418-x

**Published:** 2015-07-29

**Authors:** Ni Chen, Meiping Ren, Rong Li, Xin Deng, Yongjie Li, Kai Yan, Lamei Xiao, Yan Yang, Liqun Wang, Mao Luo, William P. Fay, Jianbo Wu

**Affiliations:** Drug Discovery Research Center, Sichuan Medical University, Luzhou, Sichuan People’s Republic of China; Department of Medicine, University of Missouri School of Medicine, Columbia, MO USA

**Keywords:** Bevacizumab, Cancer, Plasminogen activator inhibitor 1, VEGF-A, Venous thromboembolism

## Abstract

**Background:**

An increased incidence of venous thromboembolism (VTE) is associated with anti-vascular endothelial growth factor (VEGF) treatment in cancer. However, the mechanism underlying this effect remains elusive. In this study, we examined the effect of bevacizumab, a humanized monoclonal antibody against VEGF-A, on VTE in a murine xenograft A549 cell tumor model.

**Methods:**

Inferior vena cava stenosis model and FeCl_3_-induced saphenous vein thrombosis model were performed in a mouse xenograft models of human lung adenocarcinoma.

**Results:**

We found that treatment with bevacizumab significantly increased the thrombotic response to inferior vena cava obstruction and femoral vein injury. Plasminogen activator inhibitor (PAI-1) expression in tumors, plasma, and thrombi was significantly increased by bevacizumab. However, bevacizumab did not enhance VTE in PAI-1-deficient mice, suggesting that PAI-1 is a major mediator of bevacizumab’s prothrombotic effect. VEGF inhibited expression of PAI-1 by A549 cells, and this effect was neutralized by bevacizumab, suggesting that bevacizumab increases PAI-1 expression in vivo by blocking the inhibitory effect of VEGF on PAI-1 expression by tumor cells. Pharmacological inhibition of PAI-1 with PAI-039 blocked bevacizumab-induced venous thrombosis.

**Conclusion:**

Collectively, these findings indicate that PAI-1 plays a role in VTE associated with antiangiogenic therapy and the inhibition of PAI-1 shows efficacy as a therapeutic strategy for the prevention of bevacizumab-associated VTE.

## Background

Numerous clinical studies have suggested that the incidence of thromboembolic complications is further increased in cancer patients treated with antiangiogenic agents [1]. Experiments involving have shown that treatment via the inhibition of VEGF signaling significantly inhibits the resolution of venous thrombi, which could lead to persistent venous obstruction and, possibly, thrombus extension [2–4].

Bevacizumab, a recombinant humanized monoclonal neutralizing antibody against VEGF that has shown benefits in the treatment of many types of malignancy, including colorectal cancer, non-small cell lung cancer (NSCLC), renal cell carcinoma, and breast cancer, has been associated with an increased risk of serious venous thromboembolic events [5]. However, no study has yet assessed the effect of bevacizumab on the processes that govern the development of venous thrombosis. Plasminogen activator inhibitor-1 (PAI-1), the primary endogenous inhibitor of tissue-type plasminogen activator (tPA) and urinary-type plasminogen activator (uPA), has increasingly been associated with tumor growth, invasion, and metastasis. Increased PAI-1 expression by tumors is correlated with a poor prognosis [6, 7]. An elevated PAI-1 level may contribute to the development of thrombosis and has been associated with VTE [8, 9]. The objective of the present study was to determine the effect of bevacizumab on venous thrombosis in a xenograft mouse model of human lung tumors. Using pharmacological and genetic models of PAI-1 modulation, we examined role of PAI-1 as a mediator of bevacizumab’s prothrombotic effect, and tested whether the inhibition of PAI-1 can block bevacizumab-induced venous thrombosis.

## Results and discussion

**Fig. 1:**
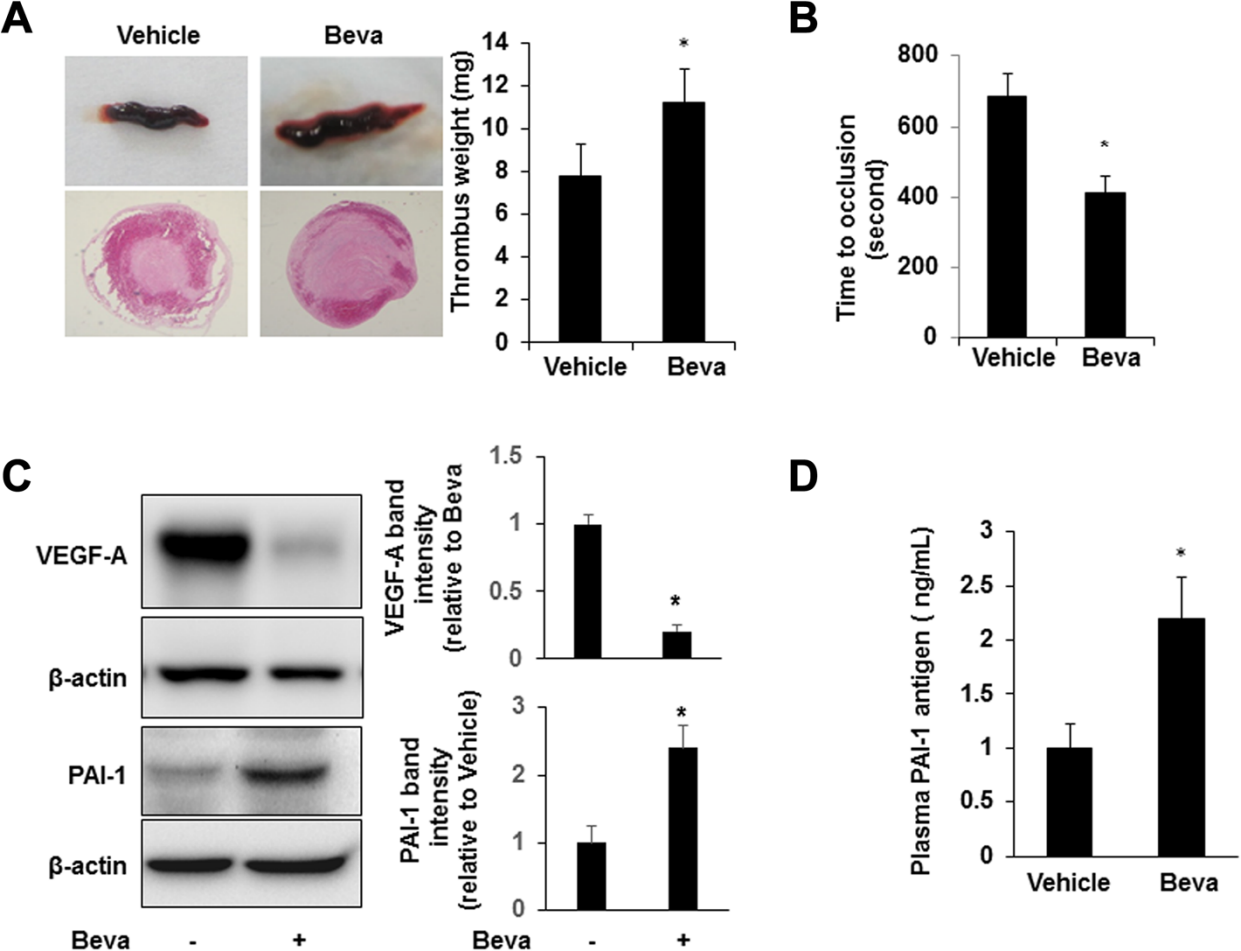
Bevacizumab promotes venous thrombosis. **a**. IVC stenosis was induced in tumor-bearing mice (n = 6/group). Three hours after ligation, mice were euthanized and the weight of the thrombus was determined. Representative whole thrombi and cross-sections of thrombi retrieved from mice treated with bevacizumab (Beva) or vehicle control are shown. *P < 0.05 vs. vehicle control. **b**. Saphenous vein thrombosis was induced using 10 % FeCl3 in control (n = 6) and A549 tumor-bearing mice (n = 6). Occlusion times were measured and are shown as the mean ± SEM. *P < 0.05 vs. the vehicle group. **c**. Tumors were excised and lysates were prepared and subjected to Western blotting to detect VEGF-A, PAI-1, and β-actin. Representative blots and densitometric analyses of 3 independent experiments are shown. *P < 0.05 vs. control. **d**. Plasma PAI-1 antigen was measured (n = 6/group); *P < 0.05 vs. the vehicle group. Beva: bevacizumab

We injected A549 cells in nude mice. After 7 weeks of tumor growth we studied venous thrombosis in tumor-bearing mice and age-matched control nude mice not bearing tumor cells. Weight of thrombi induced by inferior vena cava (IVC) partial obstruction was significantly greater in tumor-bearing mice than non-tumor-bearing controls (7.8 ± 1.5 mg *vs.* 5.6 ± 0.6 mg, respectively; n = 6/group; p < 0.01). Similarly, venous thrombosis in response to saphenous vein injury was significantly accelerated in mice bearing A549 tumors vs. negative controls (684 ± 65 sec *vs.* 828 ± 51 sec, respectively; n = 6/group; p < 0.05). These findings confirmed that our tumor model induced a prothrombotic state. In a parallel experiment, we inoculated nude mice with A549 cells. When tumors reached ~500 mm^3^ in size, bevacizumab was administered by weekly injection for up to 7 weeks, after which venous thrombosis induced by IVC stenosis was compared to the non-bevacizumab treated tumor-bearing mice described above. Bevacizumab treatment resulted in larger thrombi than those found in vehicle-treated mice (11.2 ± 1.6 mg *vs.* 7.8 ± 1.5 mg, respectively; n = 6/group; p < 0.05; Fig. 1a). In addition, bevacizumab-treated mice showed significantly shortened saphenous vein occlusion times following ferric chloride injury compared with vehicle-treated mice (411 ± 47 seconds *vs.* 684 ± 65 seconds, respectively; p < 0.05; Fig. 1b). These results indicated that treatment with bevacizumab promoted venous thrombosis in tumor-bearing mice.

**Fig. 2:**
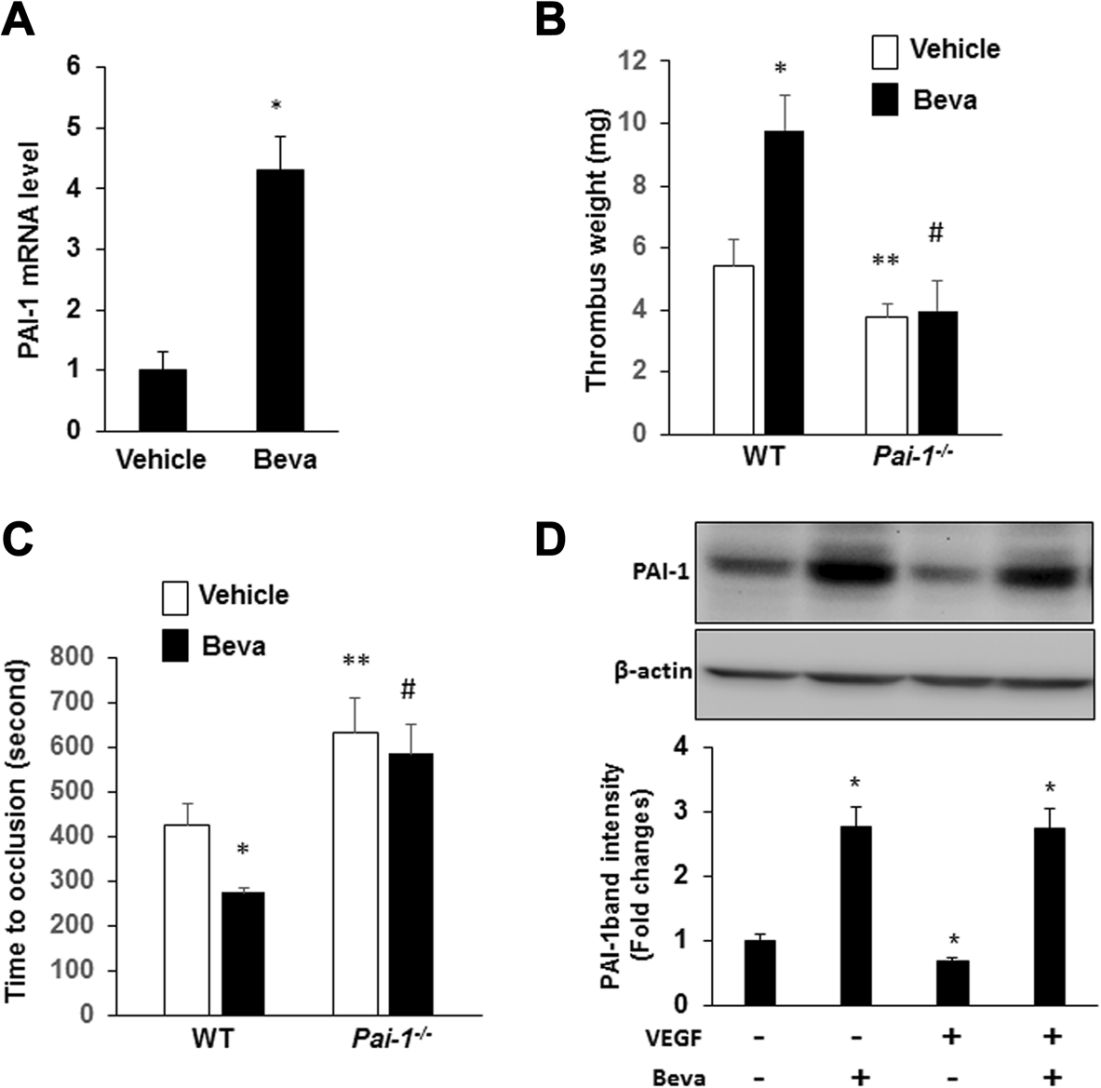
Bevacizumab promotes venous thrombosis in a PAI-1-dependent manner. **a**. The intrathrombotic gene expression of PAI-1 in the bevacizumab and vehicle groups was determined via real-time RT-PCR. All values represent mean ± SEM (n = 6/group). *P < 0.01 vs. the vehicle group. **b**. IVC stenosis was induced in WT and *Pai-1* ^*−/−*^ mice (n = 6/group). Ten days after ligation, all mice were euthanized, and the weight of the thrombus was determined. *P < 0.05 vs. the vehicle group in WT mice; **P < 0.05 vs. the vehicle group in WT mice; ^#^P < 0.05 vs. the bevacizumab group in WT mice, and ^#^P = 0.74 vs. the vehicle group in *Pai-1* ^*−/−*^ mice (n = 6/group). **c**. Saphenous vein thrombosis was induced using 10 % FeCl3 in WT and *Pai-1* ^*−/−*^ mice (n = 6/group). Occlusion times were measured and are shown as the mean ± SEM. *P < 0.05 vs. the vehicle group in WT mice; **P < 0.05 vs. the vehicle group in WT mice; ^#^P < 0.05 vs. the bevacizumab group in WT mice. **d**. A549 cells were cultured for 24 hrs in the presence and absence of VEGF (50 ng/mL) and bevacizumab (250 μg/mL), as indicated. Cell lysates were prepared and subjected to Western blotting to detect PAI-1and β-actin. Representative blot and densitometric analyses of 3 independent experiments are shown. *P < 0.05 vs. control (i.e. no VEGF or Beva)

To examine the potential role of PAI-1 in mediating the prothrombotic effect of bevacizumab, Western blot analysis using anti-human PAI-1 antibody showed that bevacizumab significantly increased tumor PAI-1 protein concentration (Fig. 1c). Bevacizumab also increased plasma concentration of tumor-derived PAI-1, which could be determined given the human origin of A549 cells and the species-specific nature of the ELISA we employed (Fig. 1d), as non-tumor-bearing mice showed no human PAI-1 in plasma. In addition, PAI-1 gene expression in the cellular component of thrombi induced by venous stasis, assessed by real-time RT-PCR, was significantly greater in bevacizumab-treated mice vs. vehicle-treated controls (Fig. 2a). At present it is difficult to state which cell type is a major target for bevacizumab to produce PAI-1 mRNA into thrombi. Potentially important source of PAI-1, besides the endothelial cells, platelets, and leukcytes, circulating tumor cells may play a role in the association between cancer and thrombosis. To assess the causal role of PAI-1 in bevacizumab-enhanced thrombosis, we administered bevacizumab or vehicle control to wild-type and *Pai1*^*−/−*^ C57Bl/6 mice. In WT mice, bevacizumab promoted venous thrombosis (Fig. 2b), indicating that bevacizumab exerts a host-dependent, prothrombotic effect, even in the absence of tumor cells. Similarly, venous thrombosis in response to saphenous vein injury was significantly accelerated in mice treated by bevacizumab vs. negative controls in WT mice (428 ± 45 sec *vs.* 276 ± 11 sec, respectively; n = 4/group; p < 0.05) (Fig. 2c). However, the prothrombotic effect of bevacizumab was lost in PAI-1-deficient mice, suggesting that PAI-1 is a major mediator of bevacizumab’s prothrombotic effect. Additional experiments, such as thromboelastogram or multiplate functional test, will be necessary to further clarify the potential importance in future studies.

To study potential mechanisms by which bevacizumab might increase PAI-1 expression, we examined the effect of VEGF on PAI-1 expression by A549 cells grown in culture. VEGF inhibited PAI-1 expression by tumor cells (Fig. 2d). Bevacizumab promoted PAI-1 expression in VEGF-treated A549 cells, consistent with bevacizumab’s VEGF-neutralizing function. Western blot analysis using anti-human VEGF antibody showed that abundant expression of VEGF in A549 tumors in vivo (Fig. 1c), and bevacizumab potently decreased VEGF protein concentration in tumors (Fig. 1c), providing an additional mechanism by which bevacizumab increases PAI-1 expression by tumor cells. As a whole, these data suggest that bevacizumab increases PAI-1 expression in vivo in tumor-bearing mice by blocking the inhibitory effect of VEGF on PAI-1 expression by tumor cells. It should also be noted that although bevacizumab has been described as binding specifically to human VEGF-A, a direct weak interaction with murine VEGF was found to be sufficient to exert this effect [10, 11]. Bevacizumab exhibited to cross-reactive with other species and has been shown to inhibit the growth of a variety of human tumor cell lines in nude mice [12, 13]. Previous studies revealed that bevacizumab does interact with mouse VEGF using three independent molecular biological assays (Western blot analysis, ELISA, and BIAcore assay), and the interaction was sufficient to induce potent inhibitory effects in murine models of corneal hemangiogenesis, lymphangiogenesis, and neovascularization and in a murine subcutaneous lung tumor model [14].

**Fig. 3:**
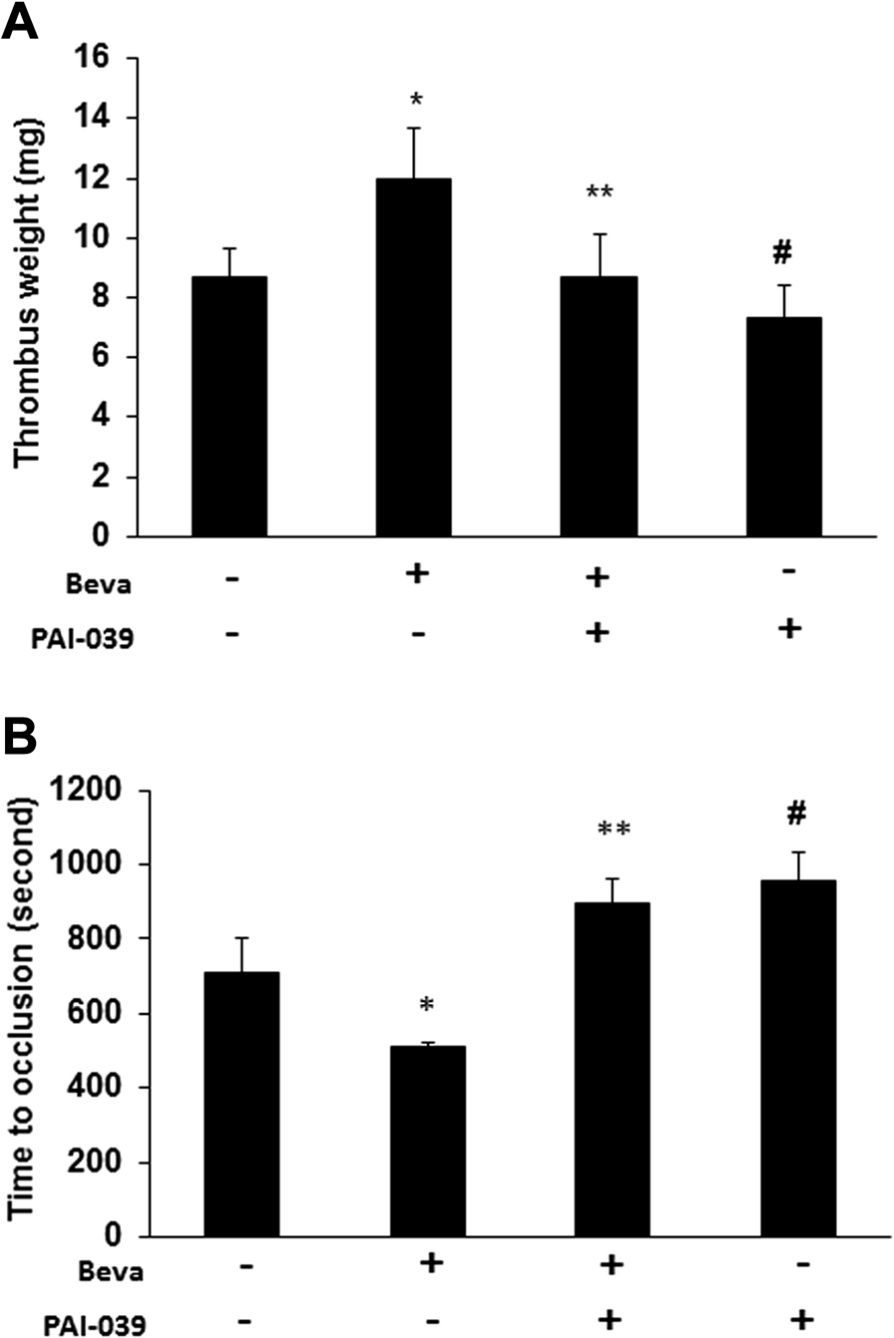
Pharmacological inhibition of PAI-1 blocks the prothrombotic effect of bevacizumab. **a**. IVC stenosis was induced in tumor-bearing mice. The mean weight of the thrombus was determined. *P < 0.05 vs. the vehicle group; **P < 0.05 vs. the beva group; ^#^P > 0.05 vs. beva + PAI-039 group (n = 6/group). **b**. Mean occlusion times were measured in a saphenous vein thrombosis model and are shown as the mean ± SEM. *P < 0.01 vs. the vehicle group; **P < 0.05 vs. the beva group; ^#^P > 0.05 vs. beva + PAI-039 group (n = 6/group). Beva: bevacizumab

**Fig. 4:**
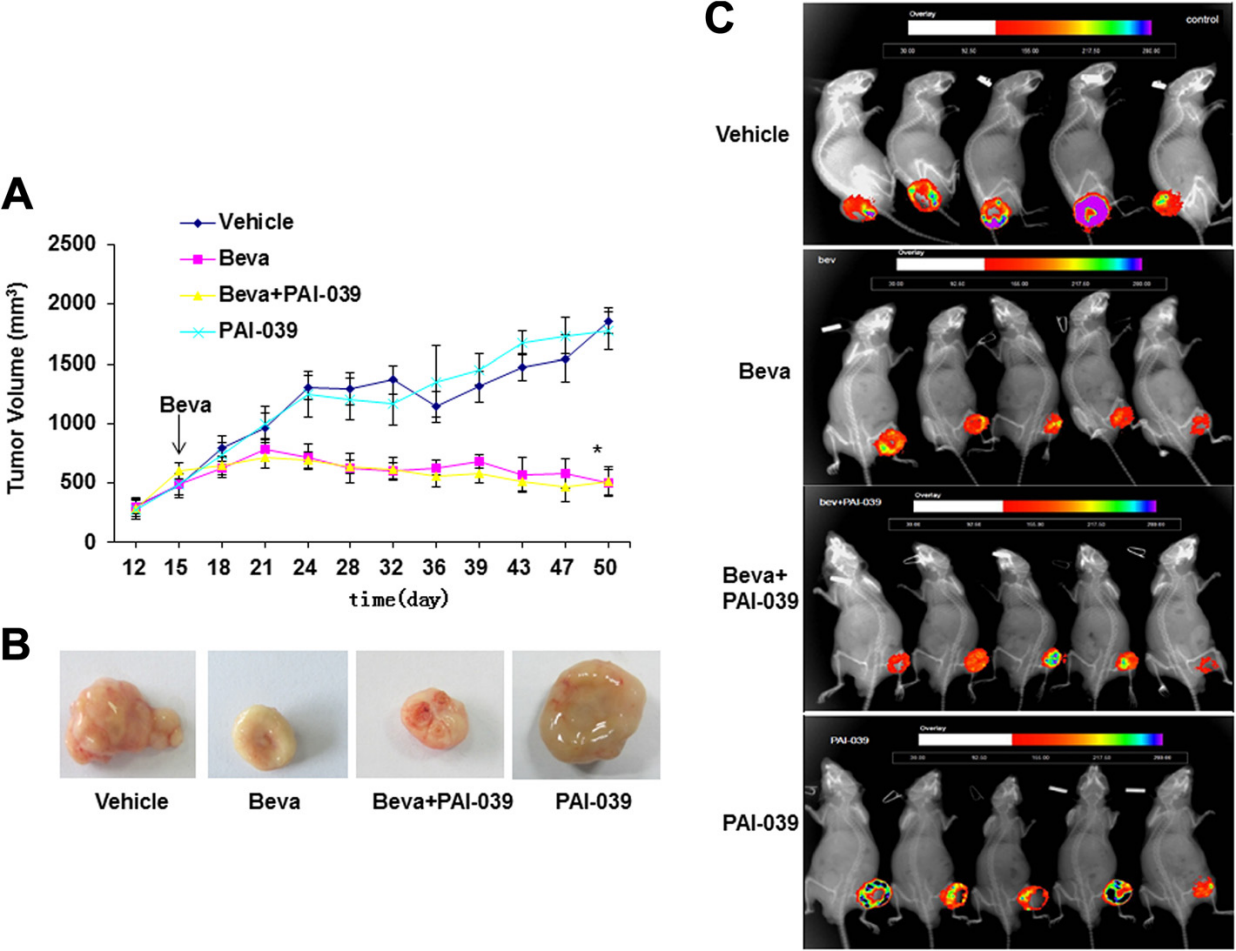
Tumor growth inhibition by bevacizumab in orthotopic A549 xenografts. **a**. A549 tumor growth curves of nude mice receiving bevacizumab (Beva; 200 μg/mouse; once weekly), PAI-039 (2 mg/kg/day), both, or vehicle control (n = 6/group). Arrow denotes first bevacizumab injection. Tumor sizes of Beva and Beva + PAI-039 groups were significantly less than those of vehicle group (P < 0.05) at 24 days and all subsequent time points, whereas differences between PAI-039 and vehicle groups were not statistically significant at any time point. **b**. Representative images of tumors retrieved at completion of treatment protocol. **c**. Bioluminescent imaging of A549-luc tumors after completion of treatment protocol. Note smaller tumor sizes in bevacizumab-treated mice

Next, we studied whether PAI-039, a specific, orally available pharmacological inhibitor of PAI-1 [15, 16], can inhibit the prothrombotic effect of bevacizumab. In tumor bearing mice, PAI-039 significantly reduced the weight of thrombi induced by IVC partial obstruction (Fig. 3a) and delayed the time to formation of occlusive thrombosis after saphenous vein injury compared to vehicle-treated controls (896 ± 67 sec *vs.* 711 ± 93 sec respectively, p < 0.05; Fig. 3b). Furthermore, bevacizumab had no significant prothrombotic effect in the presence of PAI-039, further suggesting that PAI-1 is a dominant mediator of bevacizumab’s prothrombotic effect. PAI-039 had no significant effect on tumor volume (Fig. 4a-c), suggesting that the antithrombotic effect of PAI-039 could not be explained by inhibition of tumor cell growth [17].

## Conclusions

Taken together, our data show that bevacizumab promotes venous thrombosis through the induction of PAI-1 in a mouse xenograft model of human lung carcinoma, and that pharmacological inhibition of PAI-1 may represent a useful strategy for the prevention of venous thrombosis associated with bevacizumab treatment.

## Materials and methods

### Cell culture

Human A549 lung adenocarcinoma cell line stably transfected with luciferase (A549luc) was purchased from Caliper Life sciences Corp. Cells were cultured in DMEM media supplemented with 10 % fetal calf serum (FCS), 100 U/ml penicillin and 100 U/ml streptomycin.

### Animals

Male BALB/c nude mouse mice aged 4 weeks were used for human tumor xenograft model (supplied by the Chongqing Medical University Animal Center, Chongqing, China). C57BL/6 J mice were from Jackson Labs. C57BL/6 J-congenic PAI-1-deficient (*Pai1*^−/−^) mice were a gift from Dr. Peter Carmeliet, University of Leuven, Leuven, Belgium [18]. All protocols for animal use were reviewed and approved by the Animal Care Committee of Sichuan Medical University in accordance with Institutional Animal Care and Use Committee guidelines.

### Tumor model

Male nude mice were anesthetized with ketamine/xylazine, and 1×10^6^ A549 cells were injected in the flank. Tumor volumes were measured every 3 days using Vernier calipers, and volumes were calculated using a standard formula (length × width^2^ × 0.52). When tumor volume reached ~ 500 mm^3^, mice were given bevacizumab (200 μg, administered by intraperitoneal injection) or vehicle control, after which the same dose was administered every 7 days for 50 days [19]. After completing treatment protocols, mice received D-luciferin (1.5 mg), administered by intraperitoneal injection. 3 hours later mice were euthanized and tumors were imaged by bioluminescence method using an In-Vivo Imaging System (Bruker).

### Thrombosis models

The IVC stenosis model, which reduces lumen size by approximately 90 %, was performed as described previously [20, 21], Three hours or 10 days after creation of the stenosis, mice were euthanized. Thrombi were removed from the IVC and weighed. The FeCl_3_-induced saphenous vein thrombosis model was performed as described previously [22]. Blood flow was recorded with a color laser Doppler image scanner (Moor LDI, Moor Instruments Ltd) as previously described. Flow was monitored continuously from the onset of injury until stable occlusion occurred (defined as no flow for ≥ 10 minutes) or for 60 minutes if occlusion did not occur. Occlusion time was defined as the interval between the initiation of vascular injury and the onset of stable occlusion.

### Administration of PAI-039

PAI-039 is an orally available, specific inhibitor of active PAI-1 [15]. When the average A549 tumor size reached ~ 500 mm^3^, mice were treated with PAI-039 (2 mg/kg/day; dissolved in vehicle consisting of sterile water containing 0.5 % methylcellulose and 2 % Tween 80), or vehicle control, administered for 50 consecutive days by twice daily oral gavage.

### Blood collection and plasma preparation

At time of euthanasia, whole blood was collected from the IVC into sodium citrate anticoagulant. Plasma was prepared by centrifugation at 4000 *g* for 15 minutes, followed by a clearance centrifugation of 13000 g for 2 minutes. Plasma was divided into 50 μL aliquots and frozen at −80 °C.

### Quantitative real-time PCR

Total RNA was extracted from IVC thrombus using TRIzol (invitrogen). RNA was pretreated with deoxyribonuclease I (Invitrogen Life Technologies), and SuperScript (Invitrogen Life Technologies) was used to synthesize cDNA according to the manufacturer’s recommended conditions. Each sample was analyzed in duplicate with ribosomal 18S mRNA used as controls. After amplification, the relative differences in amounts of RNA were calculated based on the 2^−Δ ΔCT^ method. The oligonucleotide sequences of PCR primers were: (1) mouse PAI-1 (GGACACCCTCAGCATGTTCA, TCTGATGAGTTCAGCATCCAAGAT); (2) 18S (CCTGGATACCGCAGCTAGGA, GCGGCGCAATACGAATGCCCC). Human VEGF (CCAGGCCCTCGTCATTG, AAGGAGGAGGGCAGAATCAT); mouse VEGF (CTCCAGGGCTTCATCGTTA, CAGAAGGAGAGCAGAAGTCC); human B-actin (GGAGGAGCTGGAAGCAGCC, GCTGTGCTACGTCGCCCTG); mouse b-actin (GGAGGAAGAGGATGCGGCA, GAAGCTGTGCTATGTTGCTCTA).

### Immunoblotting

Tumor tissues were homogenized in RIPA buffer (Sigma). Total protein concentration of the homogenates was measured with the BCA reagent. Equal amounts of protein were subjected to SDS-PAGE and transferred to polyvinylidene difluoride membranes by electroblotting. After blocking, the membranes were incubated with antibodies directed against human VEGF-A, human PAI-1, and β-actin.

### ELISA

Thrombi were retrieved from IVC 10 days after IVC ligation and homogenized in 0.3 mL of PBS (pH 7.2) containing Complete Protease Inhibitor Mixture (Roche Diagnostics). Homogenates were centrifuged at 12,000 *g* for 15 minutes. PAI-1 antigen in supernatants was measured using a mouse total PAI-1 antigen assay ELISA kit (Molecular Innovations).

### Statistical analysis

Data are presented as mean ± standard error of the mean. Experimental groups were compared by the two-tailed Student’s *t*-test or one-way analysis of variance (ANOVA).

## Abbreviations

IVC: Inferior vena cava
PAI-1: Plasminogen activator inhibitor
VEGF-A: Vascular endothelial growth factor-A
VTE: Venous thromboembolism
WT: Wild type
